# Complete Genome Sequence of *Cyanobium* sp. NIES-981, a Marine Strain Potentially Useful for Ecotoxicological Bioassays

**DOI:** 10.1128/genomeA.00736-16

**Published:** 2016-07-28

**Authors:** Haruyo Yamaguchi, Yohei Shimura, Shigekatsu Suzuki, Takahiro Yamagishi, Norihisa Tatarazako, Masanobu Kawachi

**Affiliations:** a Center for Environmental Biology and Ecosystem Studies, National Institute for Environmental Studies, Ibaraki, Japan; b Center for Health and Environmental Risk, National Institute for Environmental Studies, Ibaraki, Japan

## Abstract

*Cyanobium* sp. NIES-981 is a marine cyanobacterium isolated from tidal flat sands in Okinawa, Japan. Here, we report the complete 3.0-Mbp genome sequence of NIES-981, which is composed of a single chromosome, and its annotation. This sequence information may provide a basis for developing an ecotoxicological bioassay using this strain.

## GENOME ANNOUNCEMENT

Ecotoxicological bioassay is a widely used method for ecological risk assessment of environmental pollution. For freshwater environments, there are several standard methods using freshwater algal species to evaluate the ecotoxicity of chemicals; however, such methods are not well established for marine environments (1). To improve this situation, we are developing a method using a marine cyanobacterial strain of *Cyanobium* sp. NIES-981 because this strain demonstrates adequate growth rate and cell viability after cryopreservation. *Cyanobium* sp. NIES-981 is a unicellular, shortly rod-shaped cyanobacterium, isolated from tidal flat sands in Iriomotejima Island, Okinawa, Japan. Here, we report the complete genome sequence of *Cyanobium* sp. NIES-981, a marine strain potentially useful for ecotoxicological bioassays.

A 1-liter sample of the axenic culture was used for DNA extraction using NucleoBond AXG Columns with Buffer Set III (Macherey-Nagel). DNA sequencing was performed on the PacBio RS II sequencer (Pacific Biosciences). A 20-kb fragmented library was constructed, followed by size selection using the electrophoresis unit BluePippin (Sage Science) at 15 kb. A single library was prepared and then sequenced in a single-molecule real-time cell with P6 DNA polymerase and C4 chemistry, yielding a total of 67,150 reads. *De novo* assembly was performed by the Hierarchical Genome Assembly Process version 2.3 (2). The resulting genome comprised a single circular chromosome of 3,021,545 bp with an average genome coverage of approximately 220×. The complete genome of NIES-981 was annotated with the MicroScope platform (3), and then 3,268 protein-coding sequences, 46 tRNA genes, and three sets of rRNA genes were predicted. The G+C content of the genome was 68.62%.

The sequence of 16S rRNA was compared with that of *Cyanobium gracile* PCC 6307 and *Cyanobium* sp. PCC 7001, of which two genome sequences are available in the GenBank database (http://www.ncbi.nlm.nih.gov/Genbank/index.html), resulting in 97.31% and 98.72% similarity, respectively. The genome shares 2,194 (74%) and 2,214 (75%) of 2,965 gene families with PCC 6307 and PCC 7001, respectively.

As candidates of marine pollutants, several heavy metals derived from seabed drilling will be assumed. Thus, we subjected protein sequences of NIES-981 to a BLAST search against those of *Synechocystis* sp. PCC 6803, which is a well-studied cyanobacterium in terms of functional genes of heavy metal homeostasis and resistance (4, 5). The obtained results revealed the following genes of NIES-981 that are homologous to those of *Synechocystis* sp. PCC 6803: P_I_-type ATPases CtaA (encoded by open reading frame *slr1950*) and PacS (*sll1920*) (42% and 53% identity, respectively) for copper import; RND protein CopA (*slr6043*; 59% identity) for a copper efflux system; CopRS two-component system (*sll0789* and *sll0790*; 51% and 38% identity, respectively) for copper resistance; and ABC-type transporter MntCAB (*sll1598*, *sll1599*, and *sll1600*; 54%, 59%, and 61% identity, respectively) for high-affinity transport of manganese ions.

### Nucleotide sequence accession number.

This whole-genome shotgun project has been deposited in EMBL under the accession number LT578417. The version described in this paper is the first version.
